# Diagnostic Uncertainty in a Case of Heterotaxy Leading to Intestinal Malrotation and Volvulus

**DOI:** 10.7759/cureus.81143

**Published:** 2025-03-25

**Authors:** Maham R Sidhu, Muhammad A Hassan, Maryam M Awan, Umair Nasir

**Affiliations:** 1 Cardiology, Glenfield Hospital, Leicester, GBR; 2 Radiology, Pakistan Air Force Hospital, Islamabad, PAK; 3 Respiratory Medicine, Glenfield Hospital, Leicester, GBR

**Keywords:** heterotaxy, ladd's procedure, laprotomy, situs ambiguous, volvulus

## Abstract

We present the case of a baby with vomiting and constipation. The diagnostic tests revealed an inverted stomach, the absence of superior mesenteric vein visualization, and an elongated superior mesenteric artery, leading to the diagnosis of malrotation with situs ambiguous. Exploratory laparotomy with the Ladd's procedure was performed, and the child recovered well in the neonatal ICU. The case presented with diagnostic uncertainty in the face of vague symptoms, highlighting the need for a suspicion of malrotation and volvulus in neonates. With limited guidelines on diagnostic tests, this requires reliance on the surgeon's judgment, potentially causing delays in detecting emergent conditions like situs ambiguous leading to malrotation and volvulus.

## Introduction

Situs ambiguous or heterotaxy (HTX) syndrome or HTX disorder is a rare congenital condition with abnormal internal organs in the chest and abdomen. It can involve multiple systems and is associated with heart defects and other complications. Its exact cause is not fully understood but is believed to be related to disruptions in the early development of the embryo. It can occur sporadically or can be inherited. Diagnosis is often made through medical imaging techniques such as X-rays, ultrasounds, or CT scans. The treatment requires a multidisciplinary approach and varies among individuals. Regular medical care is essential.

## Case presentation

The case involves a baby who was born via a cesarean section at 38 weeks gestation, weighing 2.6 kg. Initially, there was no need for a stay in the neonatal intensive care unit (NICU), but on the next day, he experienced projectile vomiting with white content. The baby was then admitted to the NICU and underwent a stomach wash. He was discharged on the same day but continued to have intermittent episodes of vomiting over the following days. On the 18th day after birth, he had multiple episodes of high-volume projectile vomiting with greenish vomitus. Eventually, on the 24th day, the baby kept vomiting, was constipated, and was kept nil per os (NPO) or not given anything orally. A nasogastric (NG) tube was inserted and further investigations were performed.

**Figure 1:**
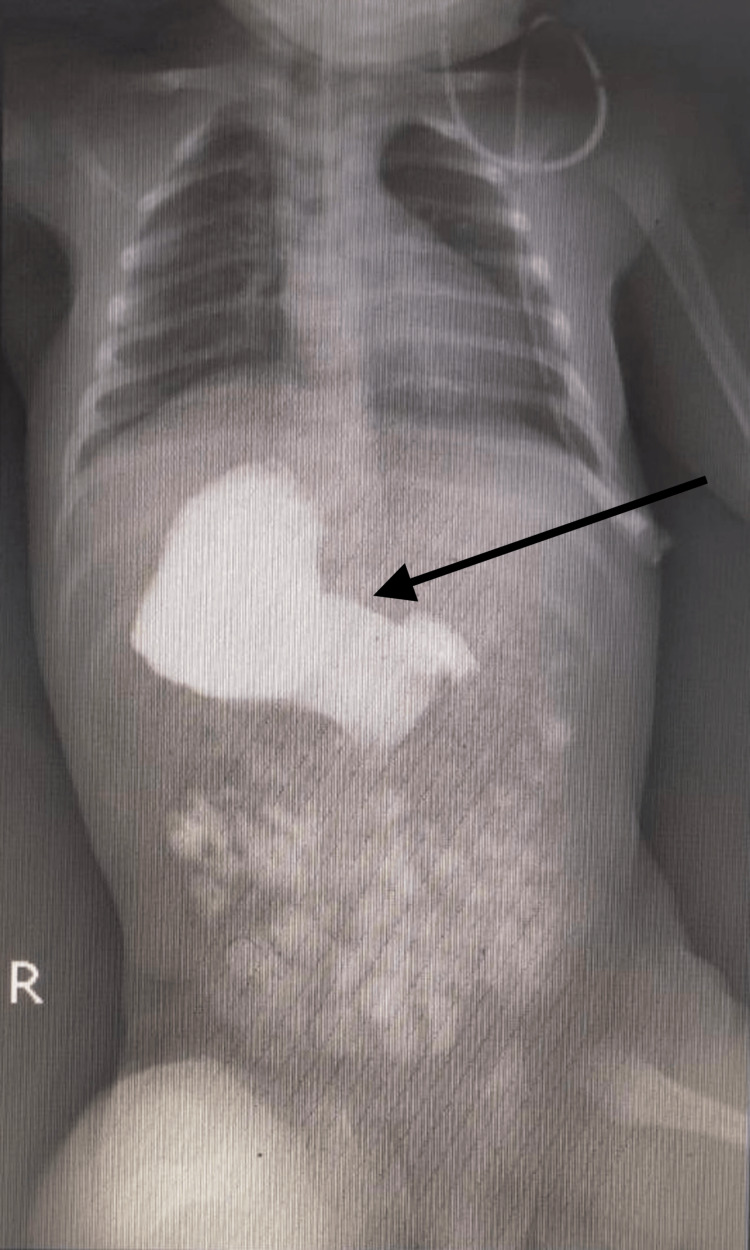
Arrow pointing to the right-sided stomach with contrast

A barium follow-through showed an inverted stomach with the greater curvature facing the right side (Fig 1). 

**Figure 2:**
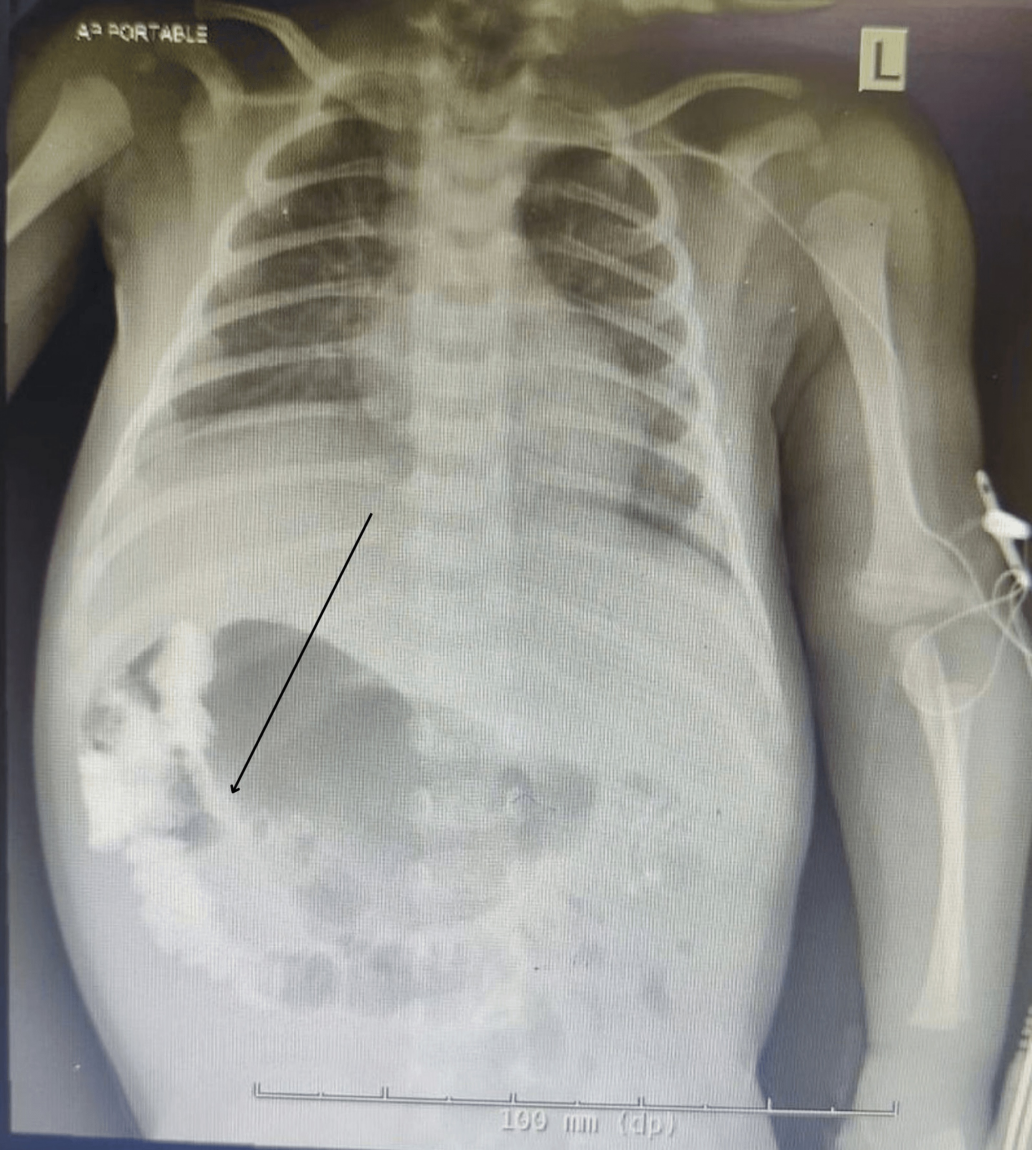
Barium follow-through shows rotated gut (arrow)

It also showed the pylorus antrum facing left of midline, raising suspicion for gastric volvulus or bowel malrotation (Fig 2).

A USG abdomen showed collapsed small bowel loops in the mid and right abdomen, elongated superior mesenteric artery coursing towards the right midline, and absence of superior mesenteric vein visualization with significant mass effect on the superior mesenteric-splenic vein confluence, raising concern for a midgut volvulus and mesenteric twist. Additional tests, such as brain ultrasound and echocardiography, showed no significant abnormalities.

The baby underwent exploratory laparotomy with a Ladd's procedure [1]. During the procedure, findings included a right-sided stomach, left-sided liver, omentum crossing the entire small bowel, left-sided duodenojejunal junction with a left-sided Ladd band, and freely mobile caecum on the right side. A left-sided transverse incision was made. The gut was delivered and retwisted, the Ladd band was released and duodenum koshered. The small bowel was placed on the left side, the large bowel on the right side, and the abdomen was closed in layers. The final diagnosis was malrotation with situs ambiguous. Following the procedure, the child was shifted to the NICU, tolerated oral nutrition, and remained there for 25 days without complications.

## Discussion

HTX is a complex congenital anomaly characterized by the abnormal arrangement of internal organs along the left-to-right axis [2]. Its incidence is estimated to range from 1 in 6000 to 1 in 20,000 live births. As per the International Society for Nomenclature of Paediatric and Congenital Heart Disease, HTX encompasses a broad range of disorders characterized by diverse patterns of organ lateralization. This variation in organ arrangement is referred to as situs ambiguous. It is distinct from situs solitus (typical intra-abdominal anatomy) or situs inversus (a complete mirror image of the intra-abdominal anatomy) [3].

Intestinal rotational abnormalities (IRA), such as malrotation, are inherent abnormalities that can result in the twisting (volvulus) of the gastrointestinal tract, causing bowel obstruction and compromised blood flow. Among patients with HTX who undergo screening for IRA, the reported incidence of malrotation ranges from 58% to 72% [4]. The intestines normally undergo a 270-degree counter-clockwise rotation around the superior mesenteric artery. The duodenojejunal junction anchors to the left upper quadrant with the ligament of Treitz, while the cecum anchors to the right lower quadrant, creating a broad mesentery that supports the superior mesenteric artery. However, in cases of classic malrotation, abnormal fixation points of the duodenojejunal junction and cecum to the retroperitoneum result in a narrow mesentery base, increasing the risk of volvulus and compromised blood supply through the superior mesenteric artery. In patients with HTX, the distance between fixation points and the breadth of the mesentery varies, leading to a lower volvulus risk compared to patients without HTX but with malrotation. 

Plain abdominal radiographs provide essential information but are usually nonspecific. Some neonates with malrotation were reported to have a normal gas pattern, thus delaying the diagnosis. The presence of a double bubble sign (air collection in the stomach and duodenum) with sparse distal air often indicates volvulus. However, one study revealed that this characteristic radiographic appearance was observed in only 65% (13 out of 20 patients) of individuals with malrotation, and its predictive accuracy for volvulus was only 62% (eight out of 13 patients) [5]. Upper gastrointestinal (UGI) contrast examination is the preferred diagnostic method for symptomatic malrotation [5], while cholescintigraphy has also been utilized but is more time-consuming. CT, MR imaging, and sonographic examination have shown good sensitivity in diagnosing intestinal malrotation by detecting abnormal positions of the superior mesenteric artery and vein.

In rare instances, where uncertainty persists despite exhausting all the imaging avenues, the decision to proceed with laparoscopy or surgery should be entrusted to the surgeon. Symptomatic patients require urgent surgery (Ladd's procedure) to prevent the death of bowel tissue. However, surgical risks are compounded by the presence of additional comorbidities associated with HTX, including congenital heart defects. Following a Ladd's procedure, there is a lifetime risk of 3-15% for bowel obstruction due to the development of new adhesions [6].

## Conclusions

This case presentation was unique due to the diagnostic uncertainty encountered by both the treating pediatrician and surgeon. The nonspecific symptoms of bilious vomiting and reluctance to feed should always raise suspicion of volvulus and malrotation in neonates. Notably, there is an absence of clear guidelines regarding the selection of diagnostic tests in such cases, which necessitates that pediatric surgeons rely on their analytical judgment and expertise to determine the appropriate timing for performing an exploratory laparotomy. While this approach may often represent the most prudent course of action, it can result in delays in diagnosing conditions such as situs ambiguous, which can be emergent and life-threatening for neonates. Furthermore, only a limited number of cases have been reported in which HTX presented with a normal spleen position but with abnormal positioning of the stomach curvature and liver. 
